# Vascular interventional strategies for recurrent nasopharyngeal carcinoma: diagnostic and therapeutic perspectives

**DOI:** 10.3389/fonc.2025.1624138

**Published:** 2025-10-24

**Authors:** Qiang Zhang, Xiang Zhai, Gang Liu, Yanguo Shang, Xiaoguang Tong, Xingwei An

**Affiliations:** ^1^ Academy of Medical Engineering and Translational Medicine, Tianjin University, Tianjin, China; ^2^ Department of Otolaryngology Head and Neck Surgery, Tianjin Huanhu Hospital, Tianjin, China; ^3^ Department of Neurosurgery, Tianjin Huanhu Hospital, Tianjin, China

**Keywords:** hemorrhage, intervention, nasopharyngeal carcinoma, recurrent, vascular interventional techniques

## Abstract

**Objective:**

This study aimed to evaluate the role of vascular interventional techniques in the management of recurrent nasopharyngeal carcinoma as part of a multidisciplinary treatment framework.

**Methods:**

A retrospective analysis was conducted on the clinical records of 14 patients with recurrent nasopharyngeal carcinoma. All participants underwent digital subtraction angiography (DSA), balloon occlusion testing (BOT), and preoperative assessments to evaluate internal carotid artery (ICA) integrity, collateral circulation, and suitability for combined arterial infusion chemotherapy in the management of ICA bleeding. Postoperative evaluations included the assessment of distal blood perfusion following ICA embolization and subsequent ICA bypass procedures.

**Results:**

DSA findings demonstrated compromised ICA involvement in three patients. Two patients with negative BOT results underwent coil embolization for hemostasis, whereas one patient with a positive BOT result received stent implantation to repair a pseudoaneurysm. This patient subsequently underwent an external carotid artery middle cerebral artery bypass, which restored satisfactory distal perfusion following ICA occlusion. Across all 14 patients, comprehensive treatment achieved substantial tumor regression with minimal systemic adverse effects. In addition, prompt intervention for ICA rupture and hemorrhage was effective, and no fatal complications occurred.

**Conclusion:**

Vascular interventional methods provided critical diagnostic and therapeutic value in the management of recurrent nasopharyngeal carcinoma. These approaches enabled the identification of tumor-feeding arteries, comprehensive assessment of vascular anatomy and distal perfusion, and mitigation of the risk of life-threatening hemorrhage. Their application established a secure foundation for subsequent therapeutic interventions targeting recurrent disease.

## 1 Introduction

Primary nasopharyngeal carcinoma is associated with a substantial risk of local or regional recurrence following radical radiotherapy, with recurrence rates estimated at 10–15% (1–4). The management of recurrent nasopharyngeal carcinoma remains clinically challenging due to limited therapeutic efficacy and the complexity of addressing post-treatment complications, including vascular stenosis in the head and neck region, carotid artery rupture, hemorrhage, and systemic adverse effects (5–7). Vascular interventional procedures like digital subtraction angiography (DSA), covered stent placement, vascular embolization, and arterial infusion chemotherapy have demonstrated their clinical use in both the diagnosis and management of recurrent disease, particularly in mitigating life-threatening hemorrhagic events during treatment. The aim of this study was to assess the clinical relevance of vascular interventional techniques in the diagnosis and treatment of recurrent nasopharyngeal carcinoma, based on a retrospective analysis of clinical data from 14 patients who underwent these interventions.

## 2 Materials and methods

### 2.1 General data

The clinical records of 14 patients who underwent vascular interventional treatment for recurrent nasopharyngeal carcinoma at the Department of Otorhinolaryngology–Head and Neck Surgery, Huanhu Hospital, Tianjin, between July 2018 and April 2022 were collected.

Inclusion criteria were as follows: (1) histologically confirmed local or regional recurrent nasopharyngeal carcinoma following radical radiotherapy (≥ 70 Gy); (2) contrast-enhanced magnetic resonance imaging (MRI) and positron emission tomography/computed tomography (PET-CT) confirming rT0-4N0-3M0 disease; and (3) indication for vascular interventional evaluation due to suspected carotid artery invasion or intractable tumor-associated bleeding.

Exclusion criteria were: (1) the presence of distant metastases requiring systemic therapy alone; (2) recurrence localized to the skull base without vascular involvement; and (3) an expected survival of less than 3 months.

The study cohort consisted of 13 male and 1 female patient, with a mean age of 52.71 ± 13.60 years (range: 32–75 years). The follow-up duration ranged from 5 months to 4 years. All patients had previously received radical radiotherapy, with a primary dose of 70 Gy administered (8). Tumor recurrence was identified between 1.5 and 11 years after the completion of radiotherapy, with a mean interval of 7 years.

The most frequently reported clinical symptoms included blood-tinged nasal mucus, epistaxis, dysphagia, choking, nasal obstruction, headache, tinnitus, diplopia, and the presence of a nasopharyngeal mass. All patients underwent nasopharyngeal pathological biopsy, as well as head and neck MRI and whole-body PET/CT to evaluate recurrence or distant metastasis and to confirm staging.

Detailed data regarding recurrent nasopharyngeal carcinoma are presented in **Table 1**. All diagnostic and therapeutic procedures were conducted in accordance with the principles of the Declaration of Helsinki, and informed consent was obtained from all patients.

**Table 1:** Clinical Data of 14 Patients with Recurrent Nasopharyngeal Carcinoma.

Case no.	Gender	Age	Tumor staging	Radiotherapy dose	Recurrence pattern	ICA involvement	Treatment process	Treatment outcome	Follow-up time (months)	DSA results
1	Male	75	rT3N0M0	70Gy	Local	The tumor is adjacent to ICA.	DSA + Surgery	CR	6	No abnormalities were observed in the imaging of the bilateral internal carotid arteries, vertebral arteries, external carotid arteries, or their branches. No aneurysms or vascular malformations were detected.
2	Male	68	rT3N0M0	70Gy	Local	The tumor is adjacent to ICA.	DSA + Radiotherapy	PR	35	No abnormalities were observed in the imaging of the bilateral internal carotid arteries, vertebral arteries, external carotid arteries, or their branches. No aneurysms or vascular malformations were detected.
3	Male	67	rT2N0M0	70Gy	Local	The tumor is adjacent to ICA.	DSA + Arterial Infusion Chemotherapy	PR	18	The tumor-feeding arteries were identified as the ascending pharyngeal artery of the left external carotid artery and the internal maxillary artery branches.
4	Male	64	rT3N0M0	70Gy	Local	The tumor is adjacent to ICA.	DSA + Surgery	CR	24	No abnormalities were observed in the imaging of the bilateral internal carotid arteries, vertebral arteries, external carotid arteries, or their branches. No aneurysms or vascular malformations were detected.
5	Male	59	rT4N1M0	70Gy	Local	The tumor encases the ICA.	DSA + Surgery	PR	26	No abnormalities were observed in the imaging of the bilateral internal carotid arteries, vertebral arteries, external carotid arteries, or their branches. No aneurysms or vascular malformations were detected.
6	Male	58	rT4N0M0	70Gy	Local	The tumor encases the ICA.	DSA + Chemoradiotherapy	PR	11	No abnormalities were observed in the imaging of the bilateral internal carotid arteries, vertebral arteries, external carotid arteries, or their branches. No aneurysms or vascular malformations were detected.
7	Male	54	rT3N0M0	70Gy	Local	The tumor encases the ICA.	DSA + Surgery + ICA Embolization + Immune Targeting	CR	11	The initial DSA imaging revealed no abnormalities. The balloon occlusion test was negative. The second DSA revealed local contrast agent extravasation at the clival segment of the left internal carotid artery, which was then embolized. The distal collateral circulation was well maintained.
8	Female	54	rT4N1M0	70Gy	Local	The tumor encases the ICA.	DSA + Immune Therapy + Chemotherapy	PR	16	No abnormalities were observed in the imaging of the bilateral internal carotid arteries, vertebral arteries, external carotid arteries, or their branches. No aneurysms or vascular malformations were detected.
9	Male	50	rT3N0M0	70Gy	Local	The tumor is adjacent to ICA.	DSA + Arterial Infusion Chemotherapy	PR	41	The tumor-feeding arteries were identified as the ascending pharyngeal artery of the left external carotid artery and the internal maxillary artery branches.
10	Male	45	rT4N0MO	70Gy	Local	The tumor encases the ICA.	DSA + Covered Stent + Immune Targeting + Bypass + Arterial Infusion Chemotherapy	PR	10	The first DSA revealed a saccular structure with an irregular shape at the petrous segment of the right internal carotid artery, measuring approximately 2.5 cm in diameter. The internal carotid artery at the petrous segment narrowed. The balloon occlusion test was positive. A covered stent was implanted. The second DSA revealed a dissecting aneurysm. The third DSA following bypass revealed that the distal branches of the middle cerebral artery were well filled. The fourth DSA detected the tumor-feeding arteries as either the ascending pharyngeal artery of the left external carotid artery or the internal maxillary artery branches.
11	Male	44	rT4N0M0	70Gy	Local	The tumor encases the ICA.	DSA + Endoscopy Combined with Microscope Surgery	CR	48	No abnormalities were observed in the imaging of the bilateral internal carotid arteries, vertebral arteries, external carotid arteries, or their branches. No aneurysms or vascular malformations were detected. Balloon occlusion tests were negative.
12	Male	34	rT3N0M0	70Gy	Local	The tumor encases the ICA.	DSA + Surgery	CR	18	No abnormalities were observed in the imaging of the bilateral internal carotid arteries, vertebral arteries, external carotid arteries, or their branches. No aneurysms or vascular malformations were detected.
13	Male	34	rT3N0M0	70Gy	Local	The tumor is adjacent to ICA.	DSA + Surgery	CR	19	No abnormalities were observed in the imaging of the bilateral internal carotid arteries, vertebral arteries, external carotid arteries, or their branches. No aneurysms or vascular malformations were detected. Balloon occlusion tests were negative.
14	Male	32	rT3N0M0	70Gy	Local	The tumor encases the ICA.	DSA + Embolization + Surgery	CR	5	DSA revealed a pseudoaneurysm on the lower wall of the lacerum segment of the left internal carotid artery. The balloon occlusion test was negative. The pseudoaneurysm and carrier artery were occluded using coil embolization of the left internal carotid artery. The collateral circulation was well-maintained.

DSA, covered stent implantation, and embolization were performed by an experienced chief vascular surgeon in collaboration with an attending vascular surgeon. Arterial infusion chemotherapy was administered by senior chief vascular surgeons from the interventional oncology department of the hospital. Vascular bypass reconstruction surgery was carried out by the neurosurgery team at the same institution.

### 2.2 Vascular intervention process

Cerebral angiography was initiated following successful puncture of the right femoral artery using the Seldinger technique, after which a 6F vascular sheath (Cordis, USA) was inserted. A 5F single-curve catheter (Boston Scientific, USA) was subsequently utilized to perform bilateral internal carotid artery (ICA) and bilateral vertebral artery angiography, enabling visualization of the course, morphology, and perfusion of intracranial arteries.

**Figure 1:**
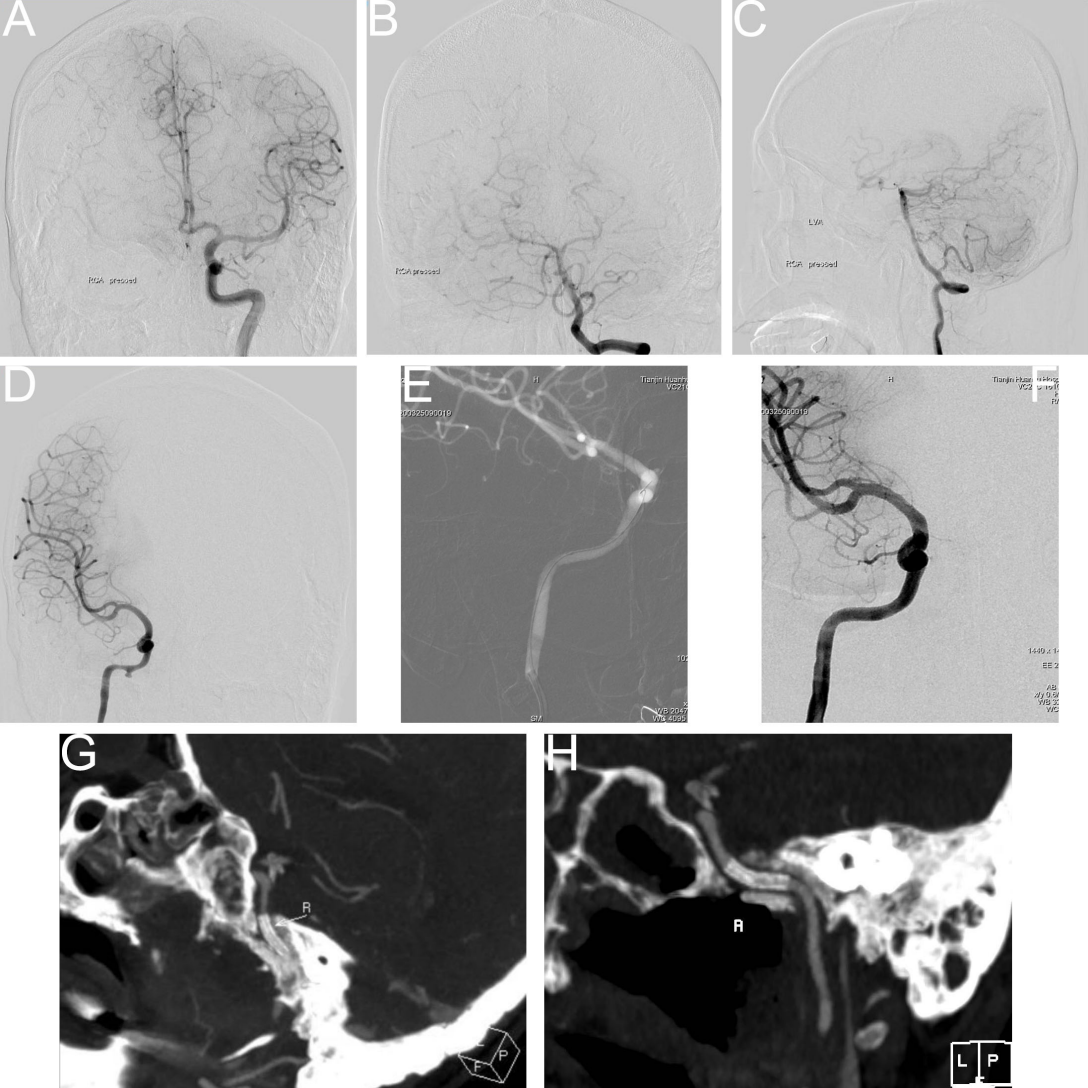
Balloon occlusion test (BOT) procedure steps. **(A)** Following balloon occlusion of the right internal carotid artery, insufficient compensatory blood flow was observed through the anterior communicating artery. **(B)** Balloon occlusion of the right internal carotid artery demonstrated limited compensatory blood flow through the posterior communicating artery. **(C, D)** Pseudoaneurysm identified at the ruptured segment of the right internal carotid artery (prior to stent implantation). **(E)** Intraoperative image during covered stent placement at the ruptured segment of the right internal carotid artery. **(F)** Post-stent implantation angiography showed unobstructed blood flow through the treated segment. **(G, H)** Computed tomography angiography (CTA) confirmed appropriate positioning of the covered stent.

During the procedure, the Matas test was first conducted (9). This involved manual compression of the affected side of the common carotid artery using the thumb from the contralateral side for several seconds prior to release. If the anterior and posterior communicating arteries were patent but demonstrated insufficient collateral perfusion, the balloon occlusion test (BOT) was generally deemed unnecessary. However, in cases where the anatomical course of the communicating arteries appeared adequate, BOT was performed for further assessment (**Figure 1**). If the BOT yielded negative results, a reduction test was carried out to objectively determine the feasibility of internal carotid artery occlusion (10, 11).

In cases with a positive BOT result, a Transend micro-slipper (Stryker, USA) was used to guide a Willis covered stent (Shanghai Minix Software Co., Ltd.) to the affected carotid artery segment harboring the aneurysm. The procedure was concluded if post-deployment cerebral angiography confirmed the disappearance of the aneurysm, satisfactory morphology of the carotid artery segment, and adequate visualization of intracranial branches. In cases where the aneurysm remained visible or recurrence was noted upon follow-up, intracranial–extracranial vascular bypass with aneurysm isolation surgery was performed (12, 13). This typically involved an anastomosis between the external carotid artery (or the lacerum segment of the ICA on the affected side) and the middle cerebral artery using a radial artery graft.

Postoperatively, cerebral angiography was repeated to evaluate the course, morphology, and perfusion of the bypass vessel. The radial artery, approximately 20 cm in length, was harvested from the contralateral upper limb by a microsurgeon. Subsequently, the neurosurgical team performed the bypass from the external carotid artery through the radial artery graft to either the petrous segment or superior promontory end of the internal carotid artery. Vascular anastomoses were typically constructed using 8–0 sutures, with 10–0 sutures used for reinforcement.

The post-stent implantation antiplatelet regimen consisted of oral administration of aspirin (100 mg) and clopidogrel (75 mg) once daily for three months. Monthly re-evaluation of blood cell counts and coagulation function (including four major coagulation parameters) was conducted throughout this period.

The initiation of arterial interventional chemotherapy for nasopharyngeal carcinoma required accurate identification of the tumor’s vascular supply. This assessment began with bilateral external carotid artery angiography, performed to delineate the vascular anatomy supplying the nasopharyngeal lesion. The primary objectives included determining if the pharyngeal ascending artery, palatine ascending artery, and maxillary artery contributed either independently or collectively to tumor perfusion, and identifying potential anastomoses between the internal and external carotid arterial systems, using the Seldinger technique.

Once the tumor-feeding arteries were confirmed, docetaxel and carboplatin, diluted in either 0.9% sodium chloride injection solution or 5% glucose injection solution, were slowly infused intra-arterially through the catheter for therapeutic administration.

Among the patients in this cohort, three demonstrated distinct pathological vessels and prominent pathological staining.

### 2.3 Efficacy assessment

Treatment efficacy was assessed based on the World Health Organization (WHO) solid tumor measurement criteria. A complete response was defined as the total disappearance of the tumor following treatment, maintained for at least 4 weeks. A partial response (PR) was defined as a reduction in tumor size > 50%, with sustained improvement for more than 4 weeks. Tumor stability or no change referred to a reduction in tumor size not exceeding 50% or an increase not exceeding 25%. Progressive disease (PD) was defined as an increase in tumor size of > 25%.

Toxicity and adverse effects were assessed based on the WHO grading standards for anticancer drug toxicity, and interventions that resulted in successful life preservation were considered effective (14).

Follow-up evaluations were conducted for all patients over a period of 5 months to 4 years. During this time, nasal endoscopy and contrast-enhanced MRI were conducted at 3-month intervals to monitor treatment response and clinical progression.

## 3 Results

All 14 patients underwent DSA to assess potential ICA damage, as well as to evaluate hemodynamic changes and signs of cerebral ischemia following ICA occlusion. This evaluation served as a critical step in guiding subsequent surgical and radio chemotherapy planning.

In 11 patients, vascular imaging revealed normal findings in the bilateral ICAs, bilateral vertebral arteries, external carotid arteries, and their respective branches, with no evidence of arterial aneurysms or vascular malformations. In 3 patients, the tumor-feeding vessels were identified as either the ascending pharyngeal artery of the external carotid artery or branches of the internal maxillary artery. Local contrast agent extravasation in the clival segment of the ICA was observed in one patient, while pseudoaneurysms were detected in the lacerum and petrous segments of the ICA in two others. Additionally, one case of dissecting aneurysm was identified after covered stent implantation.

Among the three cases involving ICA damage, two patients with negative BOT results underwent coil embolization to achieve hemostasis. The remaining patient, who had a positive BOT, was treated with stent implantation, resulting in resolution of the dissecting aneurysm. Vascular bypass was subsequently performed, involving an anastomosis between the external carotid artery and the middle cerebral artery. Post-occlusion and bypass, adequate perfusion of the distal middle cerebral artery was confirmed, with no occurrence of major complications observed (refer to **Table 1**).

All patients were followed for a duration ranging from 5 months to 4 years. During this period, endoscopic debridement of nasopharyngeal scabs was performed as needed, and appropriate therapeutic interventions were administered to all patients with residual or recurrent tumors (refer to **Table 1**). No major embolic complications, such as blindness or cerebral infarction, were reported. One patient died 6 months after the final intervention; the cause of death was attributed to cancer-related cachexia and was not associated with the interventional procedure. No treatment-related fatal complications were observed.

### 3.1 Case report

Case 1: A 45-year-old male with a history of recurrent nasopharyngeal carcinoma initially diagnosed two years after the completion of an eight-year course of radiotherapy and chemotherapy, was admitted with complaints of intermittent nasal bleeding, primarily from the right nostril, persisting for more than 10 days and described as profuse.

**Figure 2:**
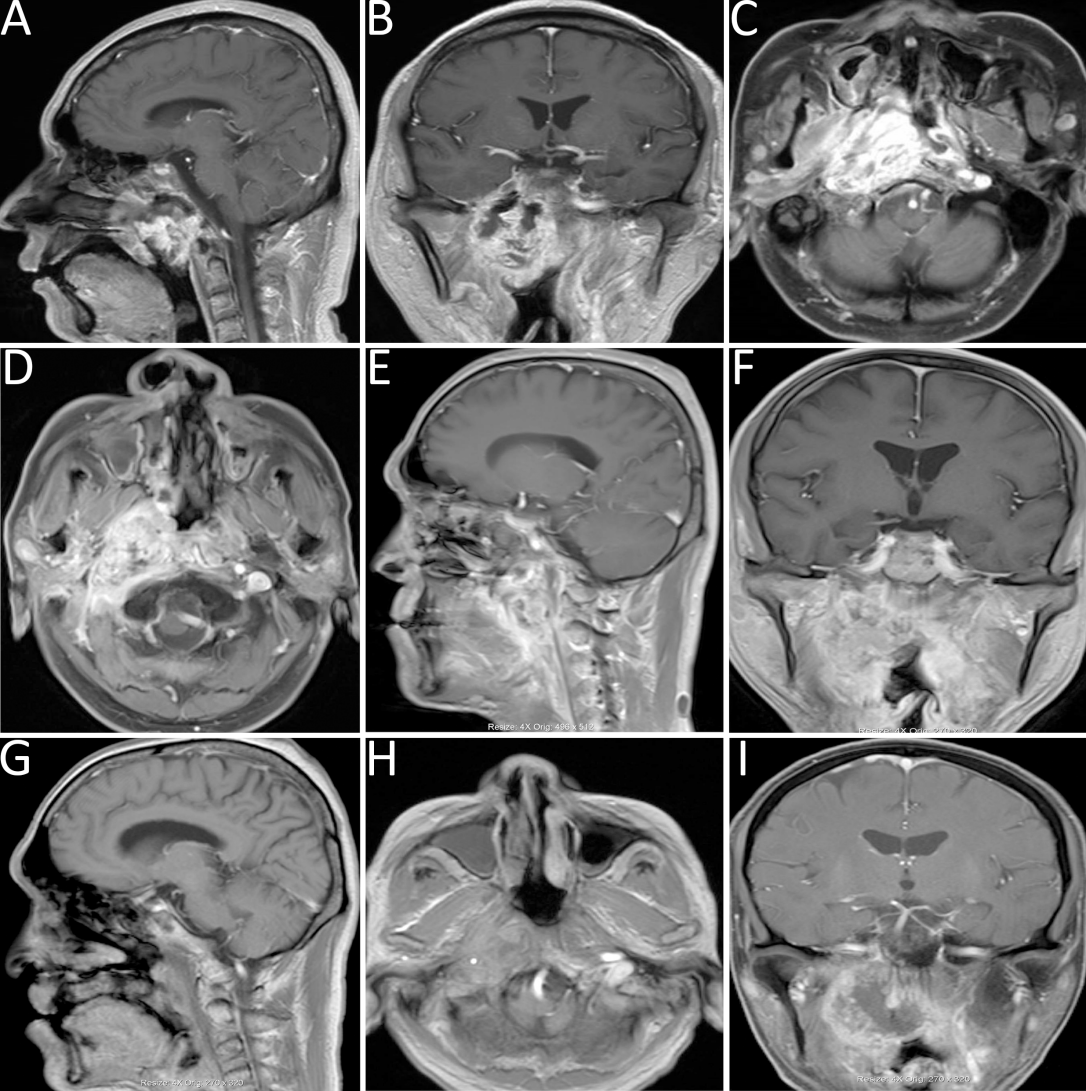
**(A-C)** Head MRI demonstrated abnormal signal intensities in the nasopharyngeal roof and right parapharyngeal space, with heterogeneous contrast enhancement and linear enhancement at the margin of the left nasopharyngeal wall. **(D-F)** MRI revealed a space-occupying lesion in the right parapharyngeal space. **(G-I)** Follow-up MRI indicated marked liquefaction and necrosis within the surgical cavity, reduction of the primary lesion, and findings consistent with a partial response (PR).

Nasal endoscopic examination revealed a nasopharyngeal mass with extensive necrotic tissue and pronounced pulsation. MRI of the head demonstrated abnormal signal intensity involving the nasopharyngeal roof and right parapharyngeal space, with areas of uneven contrast enhancement, as well as linear enhancement at the margin of the left nasopharyngeal wall (refer to **Figures 2A–C**). Given the clinical presentation and imaging findings, recurrent nasopharyngeal carcinoma accompanied by nasal bleeding was suspected.

**Figure 3:**
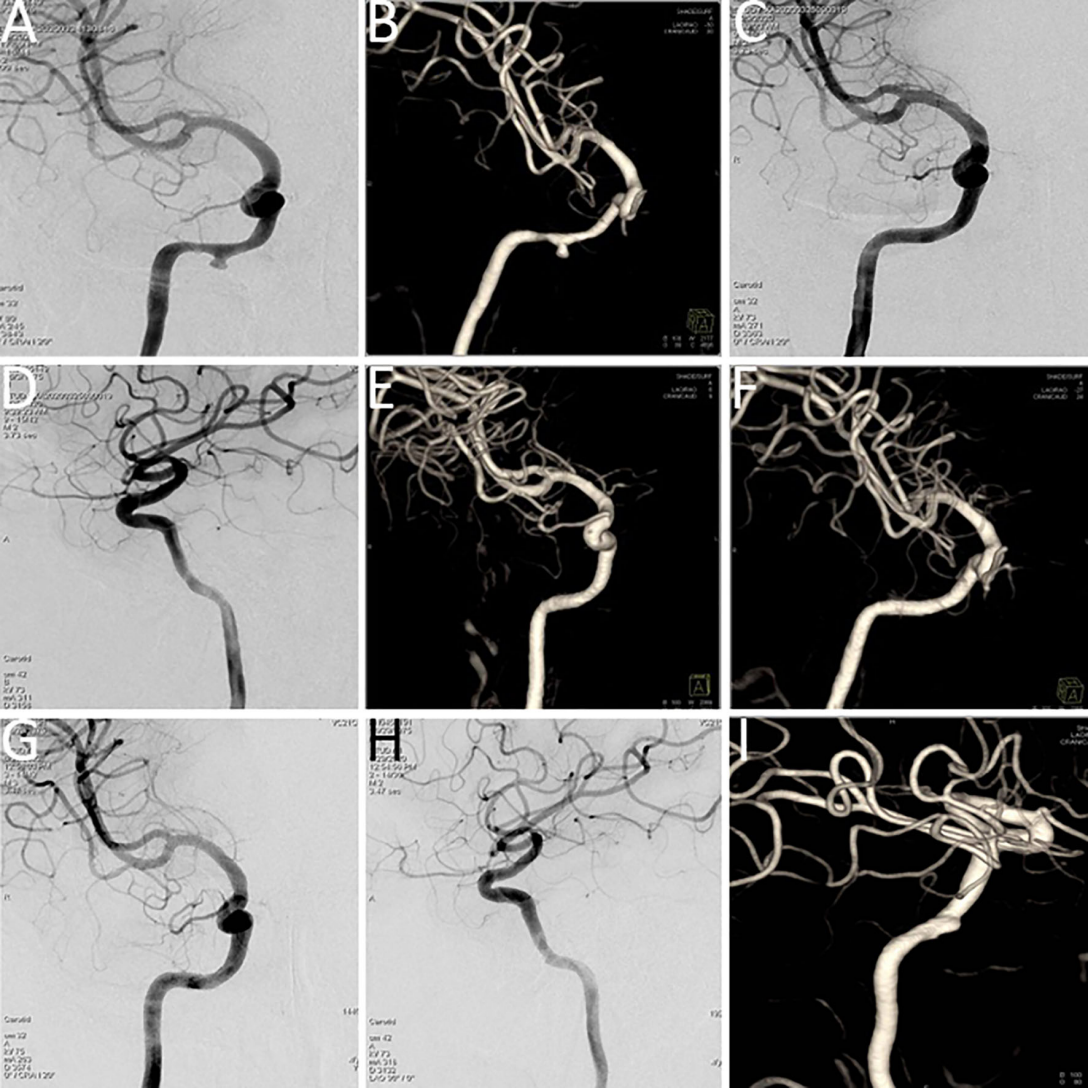
**(A, B)** Digital subtraction angiography (DSA) indicated a pseudoaneurysm in the petrous segment of the right internal carotid artery, with tumor-related narrowing; the balloon occlusion test was positive. **(C, D)** Post–covered stent implantation DSA showed disappearance of the pseudoaneurysm, restoration of normal vascular morphology, and adequate perfusion of intracranial branch arteries. **(E, F)** Vascular reconstruction after stent implantation confirmed favorable vascular morphology and intracranial branch perfusion. **(G, H)** Follow-up DSA indicated recurrence of a small aneurysm distal to the stent in the petrous segment (C2) of the right internal carotid artery. **(I)** Vascular reconstruction angiography at three months post-stent implantation demonstrated a dissecting aneurysm.

Upon admission, routine blood analysis indicated a hemoglobin level of 11.2 g/dL, raising suspicion of ICA rupture-associated hemorrhage. An urgent DSA examination confirmed the presence of a pseudoaneurysm in the petrous segment of the right ICA, with tumor-associated stenosis of the involved arterial segment. A subsequent BOT yielded a positive result (refer to **Figures 3A, B**).

Covered stent implantation was performed at the petrous segment of the right ICA, leading to the disappearance of the pseudoaneurysm. Post-procedural imaging demonstrated satisfactory vascular morphology and adequate perfusion of all intracranial branch arteries (refer to **Figures 3C–F**). Immunotherapy with sintilimab, a programmed cell death protein 1 (PD-1) immune checkpoint inhibitor, was administered every three weeks.

However, severe nasal bleeding recurred three months later. DSA and vascular reconstruction revealed septal luminal irregularities in the left carotid artery, consistent with a dissecting aneurysm (refer to **Figures 3G–I**).

**Figure 4:**
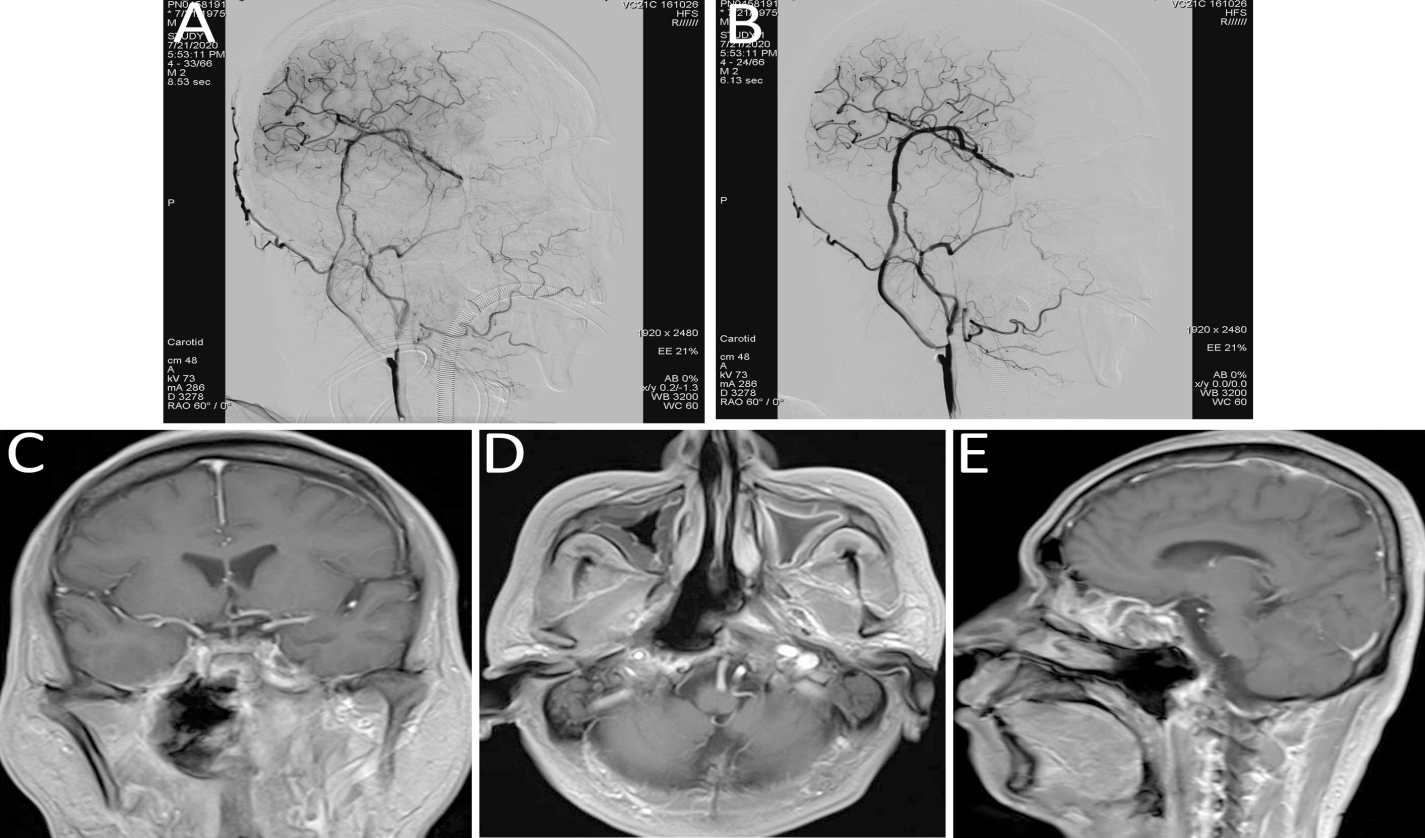
**(A, B)** Immediate post-bypass angiography confirmed patency of the bypass graft, with sufficient perfusion of the right middle cerebral artery and retrograde supply to the ophthalmic artery. No perfusion was observed in the right internal carotid artery at the cervical (C1), petrous (C2), and cavernous (C4) segments. **(C–E)** Follow-up head MRI demonstrated significant tumor shrinkage and satisfactory local disease control.

A subsequent surgical procedure was undertaken, involving the creation of a vascular anastomosis from the right external carotid artery to the middle cerebral artery using a grafted radial artery. Concurrently, the cavernous and petrous segments of the right ICA were isolated. Immediate postoperative angiography confirmed patency of the bypass, with sufficient perfusion to the right middle cerebral artery and collateral supply to the ophthalmic artery. No perfusion was observed in the cervical, petrous, and cavernous segments of the right ICA (refer to **Figures 4A, B**).

Immunotherapy and targeted treatment were continued. Follow-up MRI of the head demonstrated significant tumor shrinkage and favorable local disease control (refer to **Figures 4C–E**).

Case 2: A 49-year-old male with a history of right-sided hearing loss for one year and headaches for four months, following a five-year history of nasopharyngeal carcinoma underwent re-evaluation at this institution. Contrast-enhanced MRI indicated a space-occupying lesion in the right parapharyngeal space, consistent with recurrent nasopharyngeal carcinoma (refer to **Figures 2D–F**).

**Figure 5:**
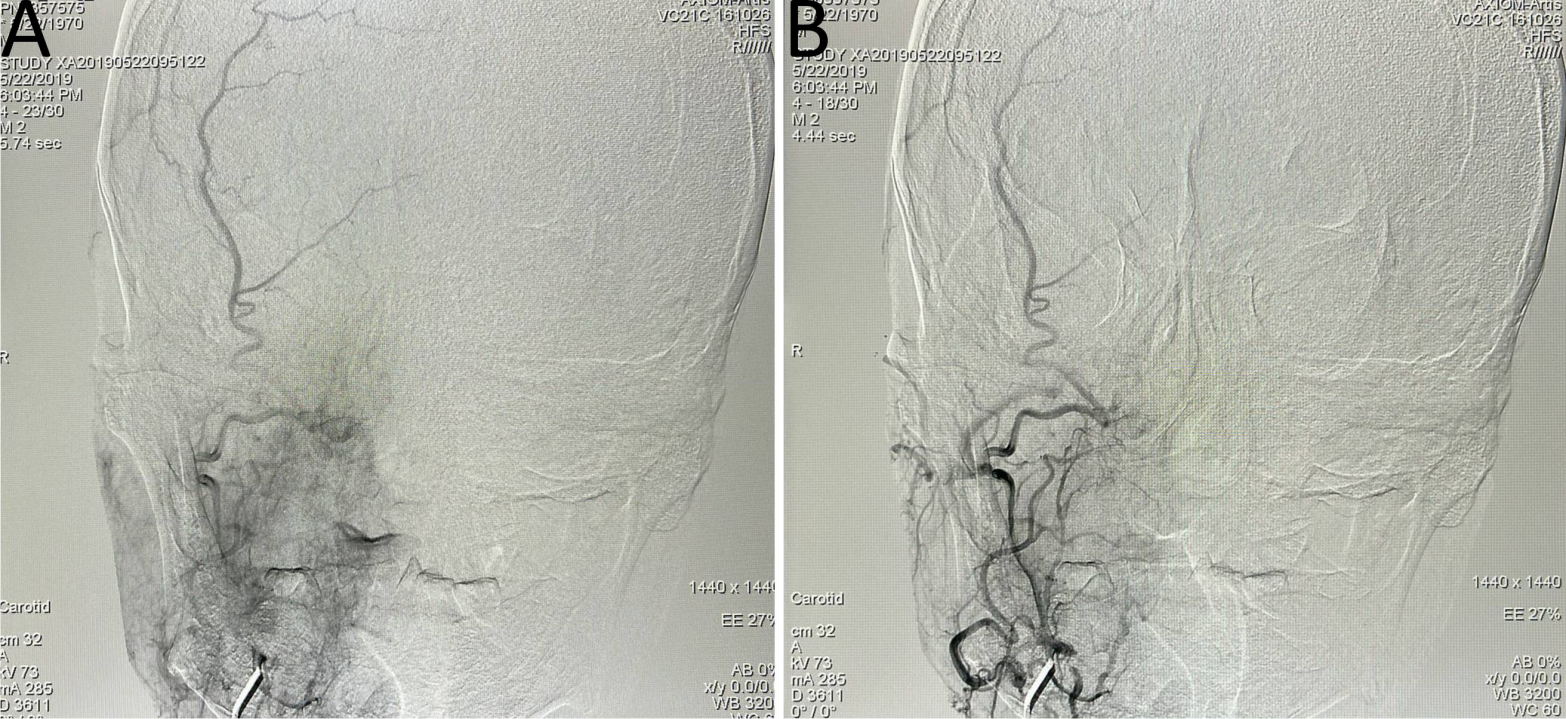
Arterial interventional chemotherapy for nasopharyngeal carcinoma targeting the ascending pharyngeal artery or internal maxillary artery branches, with intra-arterial infusion of docetaxel and carboplatin (20 mL). **(A)** ascending pharyngeal artery; **(B)** internal maxillary artery branches.

Considering the patient’s concurrent systemic conditions, arterial interventional infusion chemotherapy was selected. The chemotherapeutic agents, docetaxel and carboplatin, were diluted and administered via intra-arterial infusion (20 mL) through branches of the ascending pharyngeal artery or internal maxillary artery (refer to **Figure 5**).

Follow-up MRI demonstrated significant liquefaction and necrosis within the lesion cavity, along with a reduction in the size of the primary tumor, findings indicative of a PR (refer to **Figures 2G–I**).

## 4 Discussion

Severe nasal bleeding represents a common and critical cause of mortality in the management of advanced nasopharyngeal carcinoma and poses substantial challenges in clinical practice (15). As a result, management frequently necessitates a multidisciplinary team (MDT) approach, involving the systematic development of individualized, comprehensive treatment strategies aimed at improving therapeutic outcomes and patient quality of life (5).

Vascular risk stratification is typically addressed at the outset of weekly MDT discussions. Interventional decisions regarding carotid artery stenting or bypass surgery play a decisive role in determining if a patient is eligible for salvage surgery, re-irradiation, or systemic therapy with PD-1 inhibitors, consistent with established MDT sequencing protocols for complex aortic and vascular anomalies.

In cases involving early-stage, resectable recurrent nasopharyngeal carcinoma, surgical excision remains the preferred therapeutic modality. However, patients with recurrent disease who have previously undergone radical radiotherapy or chemotherapy frequently exhibit compromised ICAs (16). Therefore, preoperative DSA or BOT is recommended prior to initiating surgical or radio chemotherapeutic interventions.

In cases where BOT results are negative and collateral circulation is adequate, proximal ICA occlusion may be safely conducted. For patients with positive BOT results, placement of an internal carotid artery stent may be considered to preserve bilateral ICA perfusion and minimize the risk of long-term ischemic events (17). If disease progression and arterial wall compromise persist, vascular bypass surgery may be indicated.

DSA plays a key role in evaluating tumor-associated vascular anatomy and guiding the selection of appropriate treatment strategies, thereby facilitating optimal conditions for complete tumor resection.

In clinical settings, the occurrence of significant nasal bleeding exceeding 300 mL in a single episode in patients diagnosed with nasopharyngeal carcinoma substantially raises the suspicion of ICA rupture, particularly involving the petrous or lacerum segments, or of intraoperative ICA rupture (15). Several pathophysiological mechanisms contribute to this complication.

First, patients with recurrent nasopharyngeal carcinoma frequently undergo radical radiotherapy, either alone or in combination with chemotherapy. Although these treatments are directed at malignant tissue, they also cause collateral damage to surrounding normal tissues and the osseous structures of the skull base. This damage can lead to tissue necrosis and detachment, resulting in aseptic bone necrosis and exposure of the ICA wall, thereby predisposing patients to massive hemorrhage. In addition, repeated courses of radiotherapy, chemotherapy, or surgical intervention can weaken the structural integrity of the cervical arterial wall adjacent to the tumor, increasing susceptibility to rupture and subsequent bleeding.

Second, direct tumor invasion into adjacent ICAs or injury to already compromised arterial walls may result in rupture and fatal hemorrhage.

Third, following treatment, local mucosal and soft tissue injury within the nasopharynx is commonly observed. Impaired self-cleansing mechanisms and obstructed secretion drainage may predispose to secondary soft tissue infections. These infections can lead to mucosal erosion, ulceration, tissue necrosis, and detachment. Together, these factors heighten the risk of severe hemorrhagic events when combined with episodes of significant nasopharyngeal bleeding.

The initial management strategy involves the prompt implementation of endoscopic hemostasis, blood transfusion, and emergency interventions to stabilize the patient. Simultaneously, timely use of vascular interventional techniques is required to localize the bleeding source and to assess the feasibility of ICA occlusion. When occlusion is feasible, coil embolization or aneurysm clip placement at the bleeding ICA segment may be performed. If occlusion is not feasible, covered stent implantation or vascular bypass reconstruction surgery can be used to achieve hemostasis while maintaining cerebral perfusion and preventing severe complications. In this study, ICA embolization was performed in two patients, covered stent implantation in one patient, and vascular bypass reconstruction surgery in one patient. No severe complications or procedure-related mortality were reported, highlighting the effectiveness of the multidisciplinary approach in the management of ICA rupture-related bleeding.

In cases where vascular embolization is contraindicated, alternatives such as covered stent implantation or arterial bypass procedures may be considered to restore intracranial blood flow, thereby improving survival outcomes and quality of life.

For patients with nasopharyngeal ulcers and multiple systemic comorbidities who are unable to tolerate systemic chemotherapy, treatment approaches with reduced systemic toxicity are required. In this context, transcatheter arterial infusion chemotherapy was used. This method allows for higher local drug concentrations with reduced systemic adverse effects. Additionally, the elevated local chemotherapy concentration provides radio sensitizing benefits, thereby reducing the likelihood of distant metastasis or disease recurrence (18).

Among the three patients treated with arterial infusion chemotherapy, significant reductions in local tumor volume were achieved, accompanied by effective tumor control and no major complications. These findings emphasize the therapeutic efficacy, safety, and favorable tolerability of transcatheter arterial infusion chemotherapy in the management of recurrent nasopharyngeal carcinoma.

This single-center retrospective study included only 14 patients; therefore, selection bias could not be avoided, and the generalizability of the findings is limited. Prospective, multicenter trials with larger sample sizes are needed to validate both the safety and the oncologic efficacy of vascular interventional techniques in the multidisciplinary management of recurrent nasopharyngeal carcinoma. The follow-up period ranged from 5 months to 4 years (median, 22 months). Four patients were followed for fewer than 12 months, which may have led to missed late recurrences or vascular complications. Consequently, the long-term oncologic outcomes and the durability of the reconstructions remain uncertain. Future studies should incorporate prospective surveillance with a minimum follow-up of 3 years to confirm graft patency, stroke-free survival, and local disease control.

## 5 Conclusion

Vascular interventional techniques demonstrate considerable clinical value in both the diagnosis and treatment of recurrent nasopharyngeal carcinoma. These approaches facilitate delineation of the complex relationship between tumor tissue and vascular structures, identification of tumor-feeding arteries, and delivery of intra-arterial chemotherapy to reduce tumor burden. Furthermore, they play an essential role in the prevention and management of potentially life-threatening hemorrhagic complications during treatment, thereby supporting the safety and effectiveness of comprehensive therapeutic strategies for recurrent disease. However, the limitations of this study, particularly its retrospective design and small sample size, must be recognized. Prospective, controlled studies with larger cohorts are required to further validate these findings.

## Data Availability

The original contributions presented in the study are included in the article/Supplementary Material. Further inquiries can be directed to the corresponding authors.
